# Reciprocal association between depression and peptic ulcers: Two longitudinal follow-up studies using a national sample cohort

**DOI:** 10.1038/s41598-020-58783-0

**Published:** 2020-02-04

**Authors:** So Young Kim, Chanyang Min, Dong Jun Oh, Hyo Geun Choi

**Affiliations:** 1 Department of Otorhinolaryngology-Head & Neck Surgery, CHA Bundang Medical Center, CHA University, Seongnam, Korea; 2 Hallym Data Science Laboratory, Hallym University College of Medicine, Anyang, Korea; 3 Graduate School of Public Health, Seoul National University, Seoul, Korea; 4 Department of Internal medicine, Asan Medical Center, University of Ulsan College of Medicine, Seoul, Korea; 5 Department of Otorhinolaryngology-Head & Neck Surgery, Hallym University College of Medicine, Anyang, Korea

**Keywords:** Peptic ulcers, Risk factors

## Abstract

This study was aimed to explore the bidirectional association between depression and peptic ulcers. The ≥20-year-old participants of the Korean National Health Insurance Service-National Sample Cohort from 2002 to 2013 were included in the study. In study I, 30,306 depression patients were 1:4 matched with 121,224 control I participants. In study II, 127,590 peptic ulcer patients were 1:1 matched with 127,590 control II participants. The stratified Cox-proportional hazards models were used to analyse the hazard ratio (HR) of depression for peptic ulcers (study I) and of peptic ulcers for depression (study II). A total of 8.9% (2,703/ 30,306) of depression patients and 7.3% (8,896/ 121,224) of patients in the control I group had peptic ulcers (P < 0.001). The depression group had an adjusted HR for peptic ulcers that was 1.14-fold higher than that of the control I group (95% confidence interval [95% CI] = 1.09–1.19, P < 0.001). A total of 6.4% (8,144/ 127,590) of peptic ulcer patients and 3.5% (4,515/127,590) of patients in the control II group had depression (P < 0.001). The peptic ulcer group had an adjusted HR for depression that was 1.68-fold higher than that of the control II group (95% CI = 1.62–1.74, P < 0.001). Depression and peptic ulcers exhibited a bidirectional relationship.

## Introduction

Depression is a prevalent psychological disease worldwide. A meta-analysis reported an approximately 27.0% prevalence of depressive symptoms in 83 cross-sectional studies (95% confidence intervals [95% CIs] = 24.0–29.0)^1^. In Korea, about 6.7% of all age population suffered from depression (95% CI = 5.7–7.6)^2^. Depression increases the risk of several physical illnesses, such as cardiovascular disease, stroke, and diabetes^3^. The disturbances of metabolism, immune-inflammatory responses, autonomic regulation, and hypothalamic-pituitary-adrenal-axis in depression patients were suggested to be linked with the elevated risk of chronic diseases^3^. In line with this idea, depression was reported to increase the risk of a number of gastrointestinal diseases^4,5^. Several prior studies suggested a relationship between depression or psychological stress and gastrointestinal diseases^4,5^. For instance, reflux oesophagitis was related to stress (odds ratio = 1.94, 95% CI = 1.25–3.02)^4^.

Peptic ulcer disease is defined as a submucosal injury in the digestive tract, mainly in the stomach and proximal duodenum^6^. The prevalence of peptic ulcer disease is approximately 5–10%, with a decreasing tendency worldwide due to a attenuation of *Helicobacter pylori* infection and anti-acid medications^7,8^. In Korea, approximately 5.6% of the adult population has peptic ulcer disease^9^. In addition to *H. pylori* infection and the chronic nonsteroidal anti-inflammatory drug medications, the possible pathophysiological causes of peptic ulcer disease include the hypersecretion of acidic contents, dietary factors, and stress^6^. Peptic ulcers have been associated with psychological stress, schizophrenia and anxiety^10–12^. Peptic ulcer patients showed higher odds for anxiety disorders (adjusted odds ratio [AOR] = 4.41, 95% CI = 1.82–10.61) and stress (AOR = 1.11, 95% CI = 1.01–1.23)^10,12^. In addition, peptic ulcer patients had 1.47 times higher odds for depression (95% CI = 1.19–1.82)^9^. Inflammation and the disease burdens of pain, poor quality of life, and stress in peptic ulcer patients were presumed to contribute to the elevated risk of depression in peptic ulcer patients^9^. In addition to genetic factors, depression is induced by stress-related hypercortisolemia^13^. Thus, the stress from disease burden, pain, and poor quality of life could increase the risk of depression^14^.

Therefore, it can be postulated that depression and peptic ulcer diseases have a reciprocal relationship. However, few studies have evaluated the bidirectional relation between depression and peptic ulcer disease. We hypothesized that peptic ulcer disease might elevate the risk of depression and that depression elevates the risk of peptic ulcer disease. The high rate of depression in peptic ulcer patients in previous cross-sectional studies could be due to the mutual relationship between depression and peptic ulcers. To prove this hypothesis, this study investigated two independent follow-up cohort studies using control groups matched for demographic factors.

## Results

### Study I

**Figure 1:**
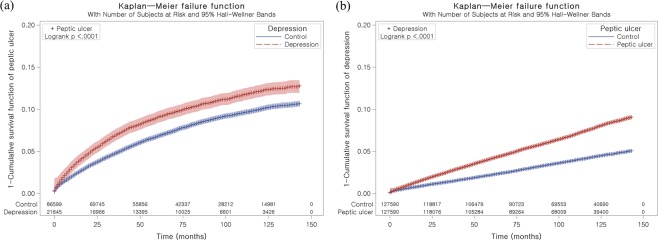
Kaplan-Meier survival analysis. (**a**) The group with depression had a higher cumulative rate of peptic ulcers than the control I group. (**b**) The group with peptic ulcers had a higher cumulative rate of depression than the control II group.

The 30.1 months (SD = 39.3) and 35.4 months (SD = 31.4) were followed in the depression group and the control I group, respectively. The rate of peptic ulcers was 8.9% (2,703/30,306) and 7.3% (8,896/121,224) in the depression group and the control group (P < 0.001, Table 1). The demographic factors were comparable between two groups (P = 1.000). The Charlson comorbidity index (CCI) was different between the depression and control I groups (P < 0.001). The depression group demonstrated 1.14 of adjusted HR for peptic ulcers (95% CI = 1.09–1.19, P < 0.001, Table 2 and Fig. 1(a)).

**Table 1:** General Characteristics of Participants.

Characteristics	Study I			Study II		
	Depression (n, %)	Control I (n, %)	P-value	Peptic ulcer (n, %)	Control II (n, %)	P-value
Age (years old)			1.000			1.000
20–24	2,133 (7.0)	8,532 (7.0)		5,815 (4.6)	5,815 (4.6)	
25–29	2,367 (7.8)	9,468 (7.8)		8,440 (6.6)	8,440 (6.6)	
30–34	2,707 (8.9)	10,828 (8.9)		11,333 (8.9)	11,333 (8.9)	
35–39	3,062 (10.1)	12,248 (10.1)		13,649 (10.7)	13,649 (10.7)	
40–44	3,127 (10.3)	12,508 (10.3)		16,126 (12.6)	16,126 (12.6)	
45–49	3,172 (10.5)	12,688 (10.5)		16,479 (12.9)	16,479 (12.9)	
50–54	2,908 (9.6)	11,632 (9.6)		14,551 (11.4)	14,551 (11.4)	
55–59	2,385 (7.9)	9,540 (7.9)		12,236 (9.6)	12,236 (9.6)	
60–64	2,163 (7.1)	8,652 (7.1)		11,348 (8.9)	11,348 (8.9)	
65–69	2,119 (7.0)	8,476 (7.0)		8,767 (6.9)	8,767 (6.9)	
70–74	1,890 (6.2)	7,560 (6.2)		5,176 (4.1)	5,176 (4.1)	
75–79	1,236 (4.1)	4,944 (4.1)		2,508 (2.0)	2,508 (2.0)	
80–84	679 (2.2)	2,716 (2.2)		883 (0.7)	883 (0.7)	
85+	358 (1.2)	1,432 (1.2)		279 (0.2)	279 (0.2)	
Sex			1.000			1.000
Male	10,436 (34.4)	41,744 (34.4)		62,105 (48.7)	62,105 (48.7)	
Female	19,870 (65.6)	79,480 (65.6)		65,485 (51.3)	65,485 (51.3)	
Income			1.000			1.000
1 (lowest)	4,807 (15.9)	19,228 (15.9)		18,381 (14.4)	18,381 (14.4)	
2	4,400 (14.5)	17,600 (14.5)		19,511 (15.3)	19,511 (15.3)	
3	5,138 (17.0)	20,552 (17.0)		23,977 (18.8)	23,977 (18.8)	
4	6,501 (21.5)	26,004 (21.5)		30,034 (23.5)	30,034 (23.5)	
5 (highest)	9,460 (31.2)	37,840 (31.2)		35,687 (28.0)	35,687 (28.0)	
Region of residence			1.000			1.000
Urban	14,247 (47.0)	56,988 (47.0)		57,870 (45.4)	57,870 (45.4)	
Rural	16,059 (53.0)	64,236 (53.0)		69,720 (54.6)	69,720 (54.6)	
CCI (score)^†^			<0.001*			<0.001*
0	2,780 (22.0)	105,613 (43.6)		45,256 (35.5)	63,137 (49.5)	
1	1,349 (10.7)	35,039 (14.5)		18,372 (14.4)	18,016 (14.1)	
2	1,757 (13.9)	31,438 (13.0)		18,753 (14.7)	14,442 (11.3)	
3	1,676 (13.2)	23,066 (9.5)		14,077 (11.0)	10,665 (8.4)	
4	1,521 (12.0)	16,808 (6.9)		10,607 (8.3)	7,722 (6.1)	
5	1,254 (9.9)	11,065 (4.6)		7,294 (5.7)	5,025 (3.9)	
≥6	2,322 (18.3)	19,492 (8.0)		13,231 (10.4)	8,583 (6.7)	
Peptic ulcer	2,703 (8.9)	8,896 (7.3)	<0.001*	127,590 (100.0)	0 (0.0)	<0.001*
Depression	30,306 (100.0)	0 (0.0)	<0.001*	8,144 (6.4)	4,515 (3.5)	<0.001*

*Chi-square test. Significance at P < 0.05.

^†^Charlson Comorbidity Index was calculated without peptic ulcer.

**Table 2:** Crude and adjusted hazard ratios (95% confidence interval) of depression for peptic ulcer (Study I), and peptic ulcer for depression (Study II).

Characteristics	Hazard ratios			
	Crude^†^	P-value	Adjusted^†‡^	P-value
Study I				
Depression	1.24 (1.19–1.30)	<0.001*	1.14 (1.09–1.19)	<0.001*
Control I	1.00		1.00	
Study II				
Peptic ulcer	1.84 (1.78–1.91)	<0.001*	1.68 (1.62–1.74)	<0.001*
Control II	1.00		1.00	

*Cox-proportional hazard regression model, Significance at P < 0.05.

^†^Stratified model for age, sex, income, and region of residence.

^‡^Adjusted model for Charlson Comorbidity index calculated without peptic ulcer.

The <40-year-old men, the <40-year-old women, and the 40–59-year-old women subgroups showed high adjusted HRs for peptic ulcer (P < 0.001, Table 3).

**Table 3:** Subgroup analyses of crude and adjusted hazard ratios (95% confidence interval) of depression for peptic ulcer according to age and sex.

Characteristics	Hazard ratios for Peptic ulcer			
	Crude^†^	P-value	Adjusted^†‡^	P-value
Age <40 years old, men (n = 18,245)				
Depression	1.30 (1.13–1.49)	<0.001*	1.17 (1.02–1.35)	0.029*
Control I	1.00		1.00	
Age <40 years old, women (n = 33,100)				
Depression	1.36 (1.22–1.51)	<0.001*	1.25 (1.12–1.39)	<0.001*
Control I	1.00		1.00	
Age 40–59 years old, men (n = 20,185)				
Depression	1.25 (1.12–1.39)	<0.001*	1.15 (1.03–1.28)	0.015*
Control I	1.00		1.00	
Age 40–59 years old, women (n = 37,775)				
Depression	1.26 (1.17–1.36)	<0.001*	1.15 (1.06–1.24)	<0.001*
Control I	1.00		1.00	
Age ≥60 years old, men (n = 13,750)				
Depression	1.01 (0.87–1.17)	0.926	0.90 (0.78–1.05)	0.167
Control I	1.00		1.00	
Age ≥60 years old, women (n = 28,475)				
Depression	1.23 (1.11–1.36)	<0.001*	1.11 (1.00–1.23)	0.053
Control I	1.00		1.00	

*Cox-proportional hazard regression model, Significance at P < 0.05

^†^Stratified model for age, sex, income, and region of residence.

^‡^Adjusted model for Charlson Comorbidity index calculated without peptic ulcer.

### Study II

The 53.2 months (SD = 37.8) and 53.4 months (SD = 38.4) were followed in the peptic ulcer group and the control II group, respectively. The rate of depression was 6.4% (8,144/127,590) and 3.5% (4,515/127,590) in the peptic ulcer group and the control II group (P < 0.001, Table 1). The demographic factors were identical between two groups (P = 1.000). The CCI was different between the peptic ulcer and control II groups (P < 0.001). The peptic ulcer group demonstrated 1.68 of adjusted HR of depression (95% CI = 1.62–1.74, P < 0.001, Table 2 and Fig. 1(b)).

The peptic ulcer group showed high adjusted HRs of depression in all subgroup analyses (P < 0.001, Table 4).

**Table 4:** Subgroup analyses of crude and adjusted hazard ratios (95% confidence interval) of peptic ulcer for depression according to age and sex.

Characteristics	Hazard ratios for depression			
	Crude^†^	P-value	Adjusted^†‡^	P-value
Age <40 years old, men (n = 37,740)				
Peptic ulcer	1.70 (1.48–1.95)	<0.001*	1.54 (1.34–1.77)	<0.001*
Control II	1.00		1.00	
Age <40 years old, women (n = 40,734)				
Peptic ulcer	1.77 (1.62–1.95)	<0.001*	1.61 (1.47–1.77)	<0.001*
Control II	1.00		1.00	
Age 40–59 years old, men (n = 59,256)				
Peptic ulcer	1.95 (1.77–2.15)	<0.001*	1.75 (1.59–1.93)	<0.001*
Control II	1.00		1.00	
Age 40–59 years old, women (n = 59,528)				
Peptic ulcer	1.89 (1.77–2.02)	<0.001*	1.72 (1.61–1.84)	<0.001*
Control II	1.00		1.00	
Age ≥60 years old, men (n = 27,214)				
Peptic ulcer	1.91 (1.70–2.14)	<0.001*	1.75 (1.56–1.96)	<0.001*
Control II	1.00		1.00	
Age ≥60 years old, women (n = 30,708)				
Peptic ulcer	1.77 (1.64–1.92)	<0.001*	1.60 (1.48–1.73)	<0.001*
Control II	1.00		1.00	

*Cox-proportional hazard regression model, Significance at P < 0.05.

^†^Stratified model for age, sex, income, and region of residence.

^‡^Adjusted model for Charlson Comorbidity index calculated without peptic ulcer.

## Discussion

Depression and peptic ulcer disease demonstrated a reciprocal relationship with one another. Depression was associated with the elevated the risk of peptic ulcer disease (adjusted HR = 1.14, 95% CI = 1.09–1.19, P < 0.001). On the other hand, peptic ulcer disease was related with the elevated the risk of depression (adjusted HR = 1.68, 95% CI = 1.62–1.74, P < 0.001). These associations were maintained in most age and sex subgroups. This is the first study to demonstrate the bidirectional relationship between depression and peptic ulcers. In addition, this study used control groups matched for demographic factors, and past medical histories were rigorously adjusted using the CCI. The previous studies also mentioned an association between depression and peptic ulcers^5,9,15^. A nationwide cohort study described that the depression patients had 1.35 times higher risk of peptic ulcer (95% CI = 1.29–1.42)^5^. Another cross-sectional study reported elevated odds of depression in peptic ulcer patients^9^. However, neither study matched the control group for the income and region of residence. Because both depression and peptic ulcers are related to the income and region of residence, these factors should be even between the study and control groups^16,17^. In addition, causality could not be concluded due to a cross-sectional study design^9^. In contrast, a cross-sectional study described no relation between depression and peptic ulcer disease^15^. The nonsignificant relationship between depression and peptic ulcer disease might have originated from the small sample of 30 peptic ulcer patients in that cohort population^15^. This study improved previous findings by using a large cohort, matched control groups, and a bidirectional study design. The bidirectional association between depression and peptic ulcer could improve the medical care of both depression and peptic ulcer patients by evaluation or management of both diseases concurrently. For instance, the medically resistant peptic ulcer patients could have untreated depression, and vice versa.

The mutual interplay between the gut and brain in immune and hormonal systems could influence the risk of peptic ulcer disease in depression patients and vice versa^18^. The immune dysfunction following the consistent activation of the hypothalamic-pituitary-adrenal axis in depression patients could elevate the risk of peptic ulcer disease. Indeed, decreases in T-cell and natural-killer-cell activities were reported in depression patients^19^. Corticotropin-releasing hormone (CRH), which is elevated in depression patients, increases gastrointestinal permeability by recruiting mast cells^20^. This hyperactivation of the hypothalamic-pituitary-adrenal axis in depression patients also indirectly influences peptic ulcer disease by disturbing the immune system^19^. Moreover, several other neuropeptides of substance P, opioids, oxytocin, and prolactin are released during stress and depression, which induces gastric mucosal hypoperfusion and gastric hypomotility^21^. Conversely, peptic ulcer disease increases the expression of neuropeptides of substance P and its receptors, thereby elevating the risk of depression^22^. In addition, peptic ulcer disease was related to immune dysfunction characterized by the downregulation of regulatory T cells and T helper cell functions^23,24^. Therefore, these hormonal and immune perturbations in peptic ulcer patients could be attributed to the occurrence of depression.

The poor diet quality induced by depression could elevate the risk of peptic ulcers. Poor diet quality was related to depression in a meta-analysis study^25^. An abnormally high-fat diet is associated with oesophageal acid exposure and gastro-oesophageal reflux, which elevate the risk of peptic ulcer disease^26,27^. Moreover, the unbalanced diet that is often associated with depression patients may influence the gut microbiota, which increases the risk of peptic ulcer disease^28^. On the other hand, chronic gastric pain and stress related to the disease burden of peptic ulcer disease could elevate the risk of depression. Chronic pain was reported to change the endocannabinoid system, which affects neurotransmission and neuroendocrine systems^29^.

The reciprocal relationship between depression and peptic ulcer disease was consistent according to age, sex, and duration of follow-up. The impact of depression on peptic ulcers and the impact of peptic ulcers on depression were maintained in short-term follow-up as well as in long-term follow-up of up to 3 years. The adjusted HRs of depression for peptic ulcers were 1.33 (95% CI = 1.24–1.43) for a follow-up period < 1 year, 1.24 (95% CI = 1.12–1.37) for 2 years, and 1.21 (95% CI = 1.07–1.36) for 3 years (P < 0.001 for each comparison, Table, Supplemental Digital Content 1). On the other hand, the adjusted HRs of peptic ulcers for depression were 1.46 (95% CI = 1.34–1.60) for a follow-up period <1 year, 1.86 (95% CI = 1.67–2.07) for 2 years, 2.02 (95% CI = 1.80–2.28) for 3 years, and 1.66 (95% CI = 1.59–1.74) for ≥4 years (P < 0.001 for each comparison, Table, Supplemental Digital Content 2).

This study used a large cohort population. In addition, several possible confounders were matched or adjusted. Both depression and peptic ulcer are associated with numerous covariates^3,10–12^. Although each possible confounder could be adjusted as covariates, the high number of variables might result in a multicollinearity of multiple variable. Therefore, we calculated CCI as single covariate in this study. This study was based on health insurance data. Thus, the disease classifications were made by physicians, which improved the accuracy of the diagnoses. On the other hand, there was a possibility of selection bias if there were differences in medical accessibility between the study and control groups. To prevent selection bias, this study included a control group matched with the study group for the income and region of residence as well as other demographic factors. In addition, the severity and medication histories of depression and peptic ulcers were not classified in this study. The depression group included bipolar disorder patients. Finally, information on the lifestyle factors of stress, dietary habits, obesity, smoking, and alcohol consumption was not available in the NHIS-NSC data. To estimate the potential influence of the lifestyle factors on depression and peptic ulcer, E-value was calculated in this study^30–32^. The E-value was 1.54 in study I and 2.75 in study II. These E-values were higher than previously reported E-values of smoking for depression (1.21)^33^ and obesity for depression (2.15)^34^, although E-value of alcohol consumption for depression was 3.41^35^. For peptic ulcer, the E-value was 1.18 of obesity, 2.08 of alcohol consumption, 2.34 of smoking, and 2.90 of use of nonsteroidal anti-inflammatory drug^36,37^. Further study considering these covariates could delineate the bidirectional association between depression and peptic ulcer after adjusting lifestyle factors.

To sum up, depression and peptic ulcers had reciprocal association.

## Materials and Methods

### Study population and data collection

The ethics committee of Hallym University approved this study (2017-I102, date approval: September 5, 2017). All methods were performed in accordance with the guidelines and regulations of the ethics committee of Hallym University. The university’s institutional review board waived the requisite for written informed consents. The study protocol conforms to the ethical guidelines of the 1975 Declaration of Helsinki as reflected in a priori approval by the institution’s human research committee.

The Korean National Health Insurance Service-National Sample Cohort (NHIS-NSC) was used for this national cohort study. The explanation on these data was detailed in our prior studies^38,39^.

### Participant selection

The individuals with depression were selected among 1,125,691 patients with 114,369,638 medical claim codes. The ICD-10 codes from F31 (bipolar affective disorder) to F33 (recurrent depressive disorder) were categorized as depression from 2002 to 2013. The depression patients who visited clinics for ≥2 times were included in this study (n = 42,370).

Peptic ulcers were categorized based on the ICD-10 codes from K25 (gastric ulcer) to K27 (peptic ulcer, site unspecified) diagnosed by the physician who conducted the endoscopy. Among these participants, the patients who were treated ≥2 times were included in this study (n = 133,349).

### Study I

**Figure 2:**
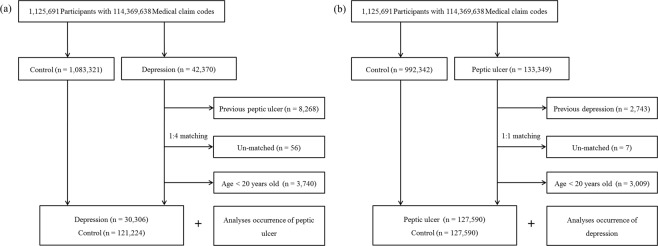
(**a**) A schematic illustration of the participant selection process used in the present study. Among a total of 1,125,691 individuals, 30,306 depression patients were matched with 121,224 control I participants based on age group, sex, income group, region of residence, and prior medical history. (**b**) Schematic illustration of the participant selection process that was used in the present study. From a total of 1,125,691 participants, 127,590 peptic ulcer patients were matched with 127,590 control II participants based on age group, sex, income group, region of residence, and past medical histories.

The control I participants who had not depression between 2002 and 2013 were matched 4:1 with the depression patients. From the total population (n = 1,083,321), the control I participants were matched with depression patients for age, sex, income, and region of residence. The control participants were assigned a random number order and then chose in consecutive order to minimize selection bias. The index date was defined as the date of the diagnosis of depression. The exclusion criteria were as follows; the control participants who died before the index date, the participants with peptic ulcers before the index date. To calculate the occurrence of depression after diagnosis of peptic ulcer, we excluded the 8,268 participants who were diagnosed as depression before the peptic ulcer. The un-matched depression patients with the control participants were removed (n = 56). In addition, the participants under 20 years old were removed (n = 3,740). The mean follow-up time was comparable between the depression (77.5 months, standard deviation [SD] = 44.0) and control I groups (78.4 months, SD = 43.9). Lastly, the 30,306 of depression participants and 121,224 control I participants were included (Fig. 2(a)). The occurrence of peptic ulcers were investigated in both the depression and control I groups.

### Study II

The control II participants who had not peptic ulcer were matched 1: 1 with peptic ulcer patients. The matching process was identical with study I. The 2,743 peptic ulcer participants who had histories of peptic ulcer before index date were excluded. The un-matched peptic ulcer patients with control II participants were excepted (n = 7). In addition, the participants who were under 20 years old were removed (n = 3,009). The mean follow-up time was comparable between peptic ulcer (98.3 months, SD = 38.3) and control II groups (97.7 months, SD = 38.6). Lastly, the 127,590 peptic ulcer patients and 127,590 control II participants were included (Fig. 2(b)). The occurrence of depression was investigated in both the peptic ulcer and control II groups.

### Variables

The age groups were divided into 14 age groups. The income groups were classified as 5 classes. The region of residence was classified as urban and rural areas.

The comorbidities were selected using ICD-10 codes. The 16 comorbidities before the index date, except for peptic ulcers, were evaluated using CCI (0 [no comorbidity] through 28 [multiple comorbidities])^40^. CCI was used as a continuous variable.

### Statistical analyses

The rate of demographic factors and comorbidities of the depression and control groups (study I) and between the peptic ulcer and control groups (study II) were analysed using a chi-square test.

The hazard ratio (HR) of depression (independent variable) for the development of peptic ulcers (dependent variable) (study I) and the HR of peptic ulcers (independent variable) for the development of depression (dependent variable) (study II) were analysed using a stratified Cox-proportional hazards model. The matched variables were stratified. Crude (simple) and adjusted (CCI) models were analysed. The 95% CI was counted. A Kaplan-Meier curve and log rank test were calculated.

To evaluate the different association according to age and sex, the participants were sub-grouped by age (20–39, 40–59, and 60+ years) and sex (men and women). Another subgroup analysis was conducted according to the follow-up periods.

Two-tailed analyses were performed. The statistical significance was considered as P < 0.05. The SPSS v. 21.0 (IBM, Armonk, NY, USA) was used for analysis.

## Supplementary information

Supplementary tables.
