# Supplementary material for: Reciprocal association between depression and peptic ulcers: Two longitudinal follow-up studies using a national sample cohort
Source: Sci Rep. 2020 Feb 4;10:1749. doi: 10.1038/s41598-020-58783-0 (PMC7000829; doi:10.1038/s41598-020-58783-0)
Supplement: Supplementary file 1 — Supplementary tables. [file 41598_2020_58783_MOESM1_ESM.pdf]

Reciprocal association between depression and peptic ulcers: Two longitudinal follow-up studies using a national sample cohort

So Young Kim, MD[1], Chanyang Min, PhD[2,3], Dong Jun Oh, MD[4], Hyo Geun Choi, MD[2,5*]

[1]Department of Otorhinolaryngology-Head & Neck Surgery, CHA Bundang Medical Center, CHA University, Seongnam, Korea

[2]Hallym Data Science Laboratory, Hallym University College of Medicine, Anyang, Korea

[3]Graduate School of Public Health, Seoul National University, Seoul, Korea

[4]Department of Internal medicine, Asan Medical Center, University of Ulsan College of Medicine, Seoul, Korea

[5]Department of Otorhinolaryngology-Head & Neck Surgery, Hallym University College of Medicine, Anyang, Korea

Running title: Depression and peptic ulcer

***Correspondence:** pupen@naver.com

Key words: depression; peptic ulcers; cohort studies; nested case-control studies; epidemiology.

Table, Supplemental Digital Content 1 Subgroup analyses of crude and adjusted hazard ratios (95% confidence interval) of depression for peptic ulcer according to follow up periods after index dates

Characteristics	Hazard ratios for Peptic ulcer			
	Crude†	P-value	Adjusted†‡	P-value
Periods ≤ 1 year				
Depression	1.48 (1.38-1.59)	<0.001*	1.33 (1.24-1.43)	<0.001*
Control	1.00		1.00	
Periods 2 year				
Depression	1.38 (1.25-1.52)	<0.001*	1.24 (1.12-1.37)	<0.001*
Control	1.00		1.00	
Periods 3 year				
Depression	1.31 (1.17-1.48)	<0.001*	1.21 (1.07-1.36)	<0.001*
Control	1.00		1.00	
Periods ≥ 4 years				
Depression	0.96 (0.89-1.04)	0.338	0.90 (0.83-0.97)	0.009*
Control	1.00		1.00	

* Cox-proportional hazard regression model, Significance at P < 0.05

† Stratified model for age, sex, income, and region of residence.

‡ Adjusted model for Charlson Comorbidity index calculated without peptic ulcer.

Table, Supplemental Digital Content 2 Subgroup analyses of crude and adjusted hazard ratios (95% confidence interval) of peptic ulcer for depression according to follow up periods after index dates

Characteristics	Hazard ratios for Depression			
	Crude†	P-value	Adjusted†‡	P-value
Periods ≤ 1 year				
Peptic ulcer	1.59 (1.46-1.73)	<0.001*	1.46 (1.34-1.60)	<0.001*
Control II	1.00		1.00	
Periods 2 year				
Peptic ulcer	2.04 (1.83-2.27)	<0.001*	1.86 (1.67-2.07)	<0.001*
Control II	1.00		1.00	
Periods 3 year				
Peptic ulcer	2.26 (2.01-2.55)	<0.001*	2.02 (1.80-2.28)	<0.001*
Control II	1.00		1.00	
Periods ≥ 4 years				
Peptic ulcer	1.82 (1.74-1.91)	<0.001*	1.66 (1.59-1.74)	<0.001*
Control II	1.00		1.00	

* Cox-proportional hazard regression model, Significance at $P < 0.05$

† Stratified model for age, sex, income, and region of residence.

‡ Adjusted model for Charlson Comorbidity index calculated without peptic ulcer.
